# Human *APP* Gene Expression Alters Active Zone Distribution and Spontaneous Neurotransmitter Release at the *Drosophila* Larval Neuromuscular Junction

**DOI:** 10.1155/2017/9202584

**Published:** 2017-07-09

**Authors:** Ekaterina A. Saburova, Alexander N. Vasiliev, Violetta V. Kravtsova, Elena V. Ryabova, Andrey L. Zefirov, Olga I. Bolshakova, Svetlana V. Sarantseva, Igor I. Krivoi

**Affiliations:** ^1^Department of General Physiology, St. Petersburg State University, St. Petersburg 199034, Russia; ^2^B.P. Konstantinov Petersburg Nuclear Physics Institute, National Research Centre “Kurchatov Institute”, Gatchina 188300, Russia; ^3^Department of Normal Physiology, Kazan State Medical University, Kazan 420012, Russia

## Abstract

This study provides further insight into the molecular mechanisms that control neurotransmitter release. Experiments were performed on larval neuromuscular junctions of transgenic *Drosophila melanogaster* lines with different levels of human amyloid precursor protein (APP) production. To express human genes in motor neurons of *Drosophila*, the UAS-GAL4 system was used. Human *APP* gene expression increased the number of synaptic boutons per neuromuscular junction. The total number of active zones, detected by Bruchpilot protein puncta distribution, remained unchanged; however, the average number of active zones per bouton decreased. These disturbances were accompanied by a decrease in frequency of miniature excitatory junction potentials without alteration in random nature of spontaneous quantal release. Similar structural and functional changes were observed with co-overexpression of human *APP* and *β-secretase* genes. In *Drosophila* line with expression of human amyloid-*β*42 peptide itself, parameters analyzed did not differ from controls, suggesting the specificity of APP effects. These results confirm the involvement of APP in synaptogenesis and provide evidence to suggest that human *APP* overexpression specifically disturbs the structural and functional organization of active zone and results in altered Bruchpilot distribution and lowered probability of spontaneous neurotransmitter release.

## 1. Introduction

Increased synthesis and accumulation of amyloid-*β*-protein (A*β*) that is produced through proteolytic processing of amyloid precursor protein (APP) by *β*- and *γ*-secretases is considered to be a major cause of Alzheimer's disease [1–3]. Data obtained on mammals indicate that APP takes part in synapse formation and in the regulation of synaptic and neuronal function, and alteration of APP expression affects cognitive and memory processes [4–6]. However, present mammalian experimental models do not allow the effects of APP and A*β* to be studied separately. One of the convenient models in this regard is *Drosophila melanogaster*. *Drosophila* endogenously express orthologue to the human *APP* (*APPL*), but it lacks the А*β* peptide region [7]. In *Drosophila*, all components of the protein complex responsible for the activity of *γ*-secretase are present and functional homolog of the *β*-secretase (BACE) has also been identified, but its activity is extremely low [8]. Therefore, in transgenic lines of *Drosophila*, it is possible to separate and study the effects of APP and A*β* independently. It has been demonstrated that APPL regulates synaptic structure and promotes synapse differentiation at the neuromuscular junction (NMJ) [9]. Experiments with expression of the human *APP* gene in *Drosophila melanogaster* demonstrated neurodegenerative changes, altered cognition and memory processes, changes in locomotion behavior, and multiple morphofunctional changes in the NMJ [10–14]. In addition, altered presynaptic function and decreased levels of the synaptic vesicle exocytosis proteins synaptotagmin and synaptobrevin were observed [12, 15].

Proteins that regulate vesicle exocytosis cluster at presynaptic active zones (AZs) that organize the synaptic release machinery to maintain and regulate synaptic transmission efficiency [16, 17]. AZs provide precise spatial and temporal control of vesicle fusion, and AZs spacing is a subject of special attention [18–21]. Notably, APP localizes to AZs and interacts with exocytosis cascade proteins, implicating APP as a potential player in neuronal communication and signaling [22–24].

Bruchpilot (Brp) is known to be the key scaffold protein of the AZ in *Drosophila*, and AZs were identified as Brp-positive puncta [19–21, 25]. This study examines the possibility that APP can be involved in the control of structural and functional presynaptic AZ organization. To test this, we analyzed the effects of human *APP* gene expression on Brp and AZs distribution as well as spontaneous quantal neurotransmitter release in the larval NMJ of transgenic *Drosophila melanogaster* lines.

## 2. Materials and Methods

### 2.1. Drosophila Strains

We used the following transgenic lines of *Drosophila melanogaster*: *UAS-APP* carries the human *APP* gene (hereinafter, APP), *UAS-BACE* carries the human *BACE* gene, double transgenic lines coexpressing human *APP* and *BACE* genes (hereinafter, APP+BACE), and UAS-A*β*42 carries the sequence of the human A*β*42 peptide (hereinafter, A*β*). Line *GAL4-D42* was used to drive transgene expression in the motor neurons and as control (hereinafter, control). *w^1118^* was used to generate outcrossed control. All transgenes and control were examined in the heterozygous state. The UAS-BACE was kindly provided by R. Reifegerste, and all other stocks were obtained from the *Drosophila* Bloomington Stock Center. During the study, the flies were kept on the standard yeast medium at a temperature of 25°C and a photoperiod of 12 h.

### 2.2. Sample Preparation and Immunohistochemistry

Third instar *Drosophila melanogaster* larvae were dissected in freshly prepared phosphate buffer (PBS) and then fixed with 4% formaldehyde (Sigma-Aldrich, United States) for 20 min, washed with PBS, blocked in blocking buffer (Visual Protein Blocking Buffer) for 1 hr, incubated with primary antibody nc82 specific to Bruchpilot (1 : 200, DSHB) overnight at 4°C and then with secondary antibodies Су-3 (1 : 200, Jackson ImmunoResearch) and with antibodies specific to HRP, conjugated with Alexa Fluor 488 (1 : 400, Jackson ImmunoResearch) for 2 hr at room temperature. Samples were mounted in VectaShield (Vector Laboratories) and visualized under a Leica DMI6000 confocal microscope equipped with a 40x, 1.3 NA objective (Leica, Germany) at 488 nm and 543 nm. Confocal images of muscle 4 NMJ containing 1b boutons were analyzed. Satellite boutons were defined as small protrusions emanating from the primary axial branch of nerve terminals. A number of confocal images of muscle 6 and 7 NMJs were also analyzed. NMJ morphology was estimated with Leica Application Suite X software (Leica, Germany). Brp clusters (and corresponding AZs) were counted manually.

The primary antibody nc82 was obtained from the Developmental Studies Hybridoma Bank, created by the NICHD of the NIH and maintained at the University of Iowa, Department of Biology, Iowa City, IA 52242.

### 2.3. Electrophysiology

Electrophysiological experiments were performed on freshly prepared third instar *Drosophila* larvae in HL3 saline (in mM: 70 NaCl, 5 KCl, 20 MgCl_2_, 2 CaCl_2_, 10 NaHCO_3_, 5 Trehalose, 115 sucrose, 5 HEPES, pH 7.2). Larvae were dissected and prepared in low Ca^2+^ (0.2 mM) HL3 saline at 4°С. After preparation, larvae were maintained in a Plexiglas chamber continuously perfused with normal HL3 saline (containing 2 mM Ca^2+^) using a BT-100 two-channel peristaltic pump (Leadfluid Co). The solution was continuously aerated with a 95% O_2_ and 5% CO_2_ gas mixture and maintained at 22°C using a CL-100 bipolar temperature controller equipped with a SC-20 heating/cooling element (Warner Instruments, USA). Spontaneous neurotransmitter release was monitored by intracellularly recording miniature excitatory junction potentials (mEJPs). Resting membrane potentials and mEJPs were recorded from muscle fibers 6 and 7 using glass microelectrodes with internal capillaries BF150-110-10, made with a P-97 microforge (Sutter Instrument Co., USA). The electrodes were filled with 3 M KCl and their resistance was about 10 mOhm. Recordings were made in junctional membrane regions within visually identified terminal branches of the motor nerve using the binocular PZMTIII microscope (WPI). The resting membrane potentials and mEJPs were amplified and digitized using the Axoclamp 900A and the Digidata 1440A (Molecular devices, USA), with automated data statistics processing (PC Clamp 10.0). Spontaneous mEJPs were recorded for 3–6 min, and the average parameters of the spontaneous events were quantified. Only recordings with stable resting membrane potential below −60 mV throughout the course of the experiment were analyzed. Peak amplitude, rise time (10% to 90% of the baseline-to-peak amplitude range), and time to decay half-amplitude of individual mEJPs were digitized and analyzed.

### 2.4. Statistical Analysis

Data are given as the mean ± SEM. Statistical significance of the difference between means was evaluated using Student's *t*-test and one-way ANOVA (OriginPro 8 software). Cumulative probability distributions were compared with the Kolmogorov-Smirnov test (GraphPad Prism 7 software).

## 3. Results

### 3.1. Human APP Gene Expression Specifically Alters Active Zone Distribution

Muscle 4 NMJs in APP and APP+BACE lines demonstrated a significant (*P* < 0.01) increase in the number of both 1b and satellite boutons per NMJ compared to the control; accordingly, total bouton number per NMJ also increased (Table 1). These data provide further evidence that human APP overexpression enhances proliferation and synaptogenesis and confirms similar observations previously obtained in *Drosophila melanogaster* [9, 12]. In conditions of direct expression of A*β* (line A*β*), the average number of boutons of both types did not differ from the control (Table 1).

**Figure 1:**
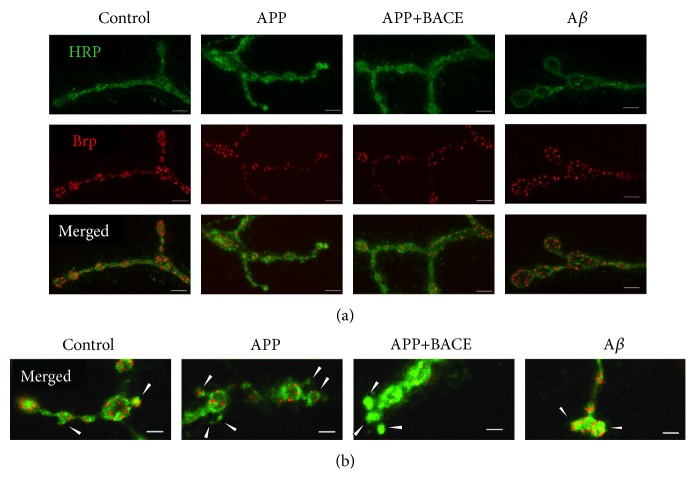
Muscle 4 neuromuscular junction. Bruchpilot and corresponding active zone distribution in synaptic boutons from transgenic *Drosophila melanogaster* larvae. (a) 1b boutons. (b) Satellite boutons (identified by arrowheads). Representative confocal images from the control, APP, APP+BACE, and А*β* lines are shown. Muscle 4 neuromuscular junctions were costained with antibodies against HRP (green channel) and the presynaptic AZ protein Brp (red channel). Scale bars, 5 *μ*m (a) and 2 *μ*m (b).

The localization of individual AZs was determined by Brp protein clusters (Figure 1). In APP and APP+BACE lines, the average number of AZs per 1b bouton was significantly (*P* < 0.01) lower than that in the control, and a similar effect was observed in satellite boutons (Table 1). Nevertheless, the total AZ number per NMJ did not differ from that in the control (Table 1), presumably due to increased synaptic bouton number that compensates AZs loss. In the A*β* line, the number of AZs per bouton did not significantly differ from that in the control (Table 1).

**Figure 2:**
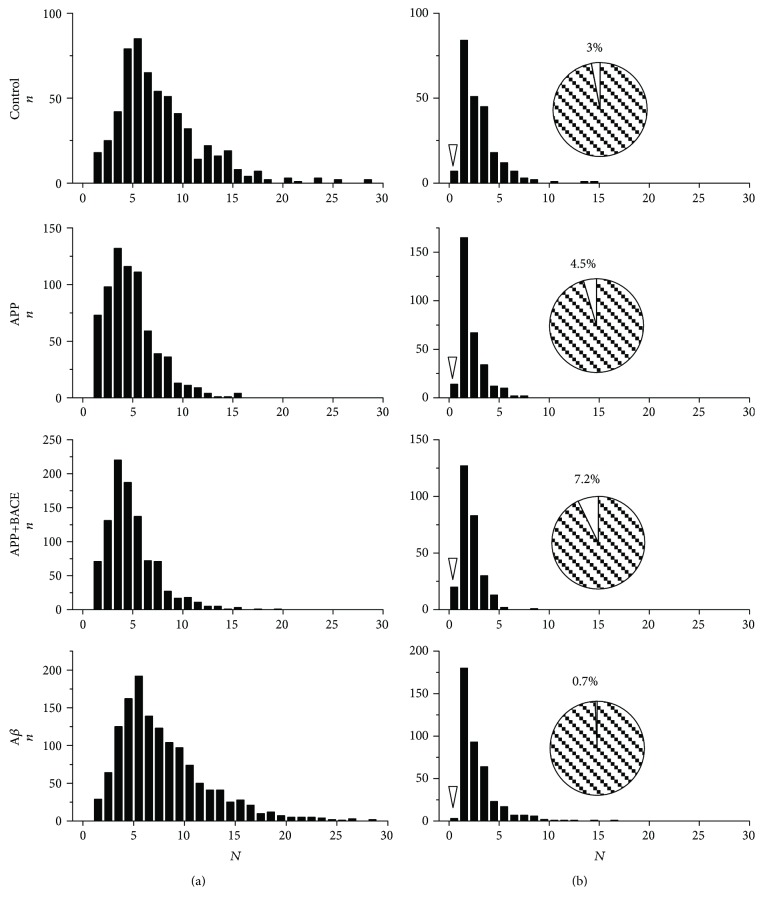
Muscle 4 neuromuscular junction. Bruchpilot cluster (active zone) distribution in individual synaptic boutons from transgenic *Drosophila melanogaster* larvae. Distributions for the control, APP, APP+BACE, and А*β* lines are shown. (a) 1b boutons. (b) Satellite boutons; arrowheads identify the boutons containing no AZs. Insets—circle charts showing percentage of satellite boutons containing no AZs. N—active zone number per single bouton.

**Figure 3:**
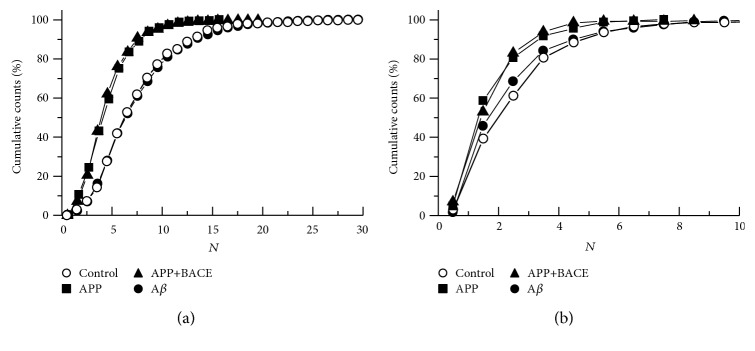
Muscle 4 neuromuscular junction. Cumulative probability of Bruchpilot cluster (active zone) distribution in individual synaptic boutons from transgenic *Drosophila melanogaster* larvae. (a) 1b boutons. (b) Satellite boutons. Control line—open circles; APP line—squares; APP+BACE line—triangles; and А*β* line—filled circles. The distribution of AZs per bouton in the APP and APP+BACE lines are significantly shifted toward fewer numbers compared to the control (*P* < 0.01, Kolmogorov-Smirnov test). In the А*β* line, cumulative probability is not significantly different from the control. N—active zone number per single bouton.

Distribution of Brp clusters (AZs) in individual 1b boutons (Figure 2(a)) and corresponding cumulative probability curves (Figure 3(a)) in APP and APP+BACE lines were significantly (*P* < 0.01, Kolmogorov-Smirnov test) shifted towards fewer numbers compared to the control. A similar effect was observed for satellite boutons (Figures 2(b) and 3(b)). In the A*β* line, AZs distributions and cumulative probability curves did not differ from the control (Figures 2 and 3).

A very small fraction of satellite boutons (3% in the control) did not contain AZs at all and presumably could be referred to ghost boutons [26]. In APP and APP+BACE lines, the number of satellite boutons containing no AZs was higher than in the control, while this effect was not observed in the A*β* line (Figure 2(b)). These data further confirm that human *APP* overexpression alters the AZ distribution.

Importantly, the enhanced synaptogenesis accompanied by the alteration of AZ distribution observed in APP and APP+BACE lines was not observed in the A*β* line. This suggests that the observed effects are specific to APP and not related to A*β* production.

**Figure 4:**
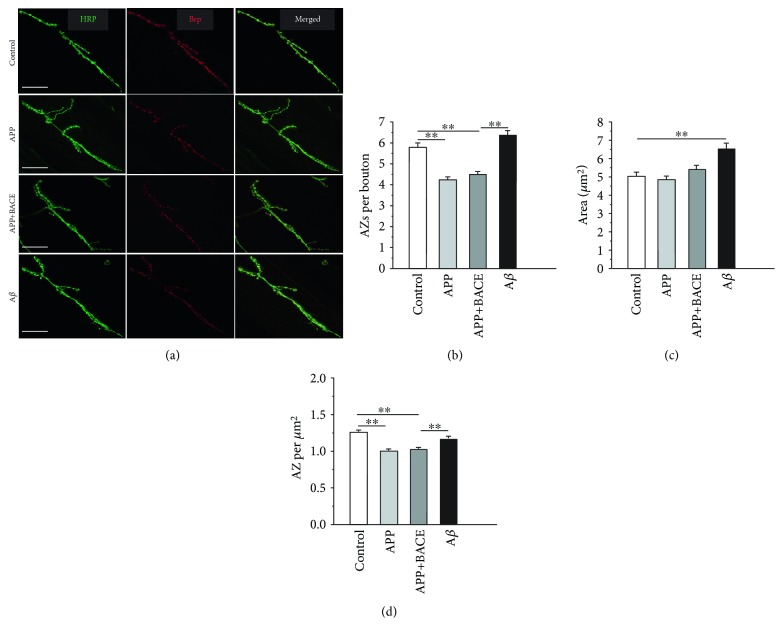
Muscle 6 and 7 neuromuscular junctions. (a) Bruchpilot and corresponding active zones distribution in synaptic boutons from transgenic *Drosophila melanogaster* larvae. Representative confocal images from the control, APP, APP+BACE, and А*β* lines are shown. Muscle 6 and 7 neuromuscular junctions were costained with antibodies against HRP (green channel) and the presynaptic AZ protein Brp (red channel). Scale bars, 25 *μ*m. (b) Average number of active zones per bouton. (c) Average boutons area, *μ*m^2^. (d) Spatial density of AZs per *μ*m^2^. Data are presented as mean ± SEM. ^∗∗^*P* < 0.01 compared to corresponding bar.

Notably, neuron-type specificity at muscle 4 NMJ compared to muscle 6 and 7 NMJs regarding *Drosophila* neurotrophins was observed [26]. To test whether the effects of APP is neuron-type specific or not, a number of confocal images of muscle 6 and 7 NMJs containing both 1b and 1s boutons were analyzed additionally (Figure 4(a)). The average number of AZs per bouton in the APP line (4.3 ± 0.1; 6 NMJ from 6 larvae) and in APP+BACE line (4.5 ± 0.1; 9 NMJ from 6 larvae) were significantly (*P* < 0.01) lower than that in the control (5.8 ± 0.2; 11 NMJ from 6 larvae). Conversely, in the A*β* line, the number of AZs per bouton (6.4 ± 0.2; 9 NMJ from 5 larvae) tend to increase compared to that in the control (Figure 4(b)).

Additionally, to test if APP affects AZ spatial density, area of each individual bouton (Figure 4(c)) and the number of AZs per corresponding bouton area were estimated. The average spatial density of AZs per *μ*m^2^ in the control line was 1.27 ± 0.02 (382 boutons), and this parameter was significantly (*P* < 0.01) lower both in APP (1.01 ± 0.02 per *μ*m^2^; 322 boutons) and in APP+BACE (1.03 ± 0.02 per *μ*m^2^; 534 boutons) lines (Figure 4(d)). Again, in the A*β* line, the spatial density of AZs (1.18 ± 0.02 per *μ*m^2^; 381 boutons) was significantly (*P* < 0.01) higher compared to APP and APP+BACE lines and was similar to the control (Figure 4(d)). These data indicate that spatial density of AZs changes in the same way as the number of AZs per bouton (Figures 4(b) and 4(d)).

### 3.2. Human APP Gene Expression Specifically Alters Spontaneous Neurotransmitter Release

**Figure 5:**
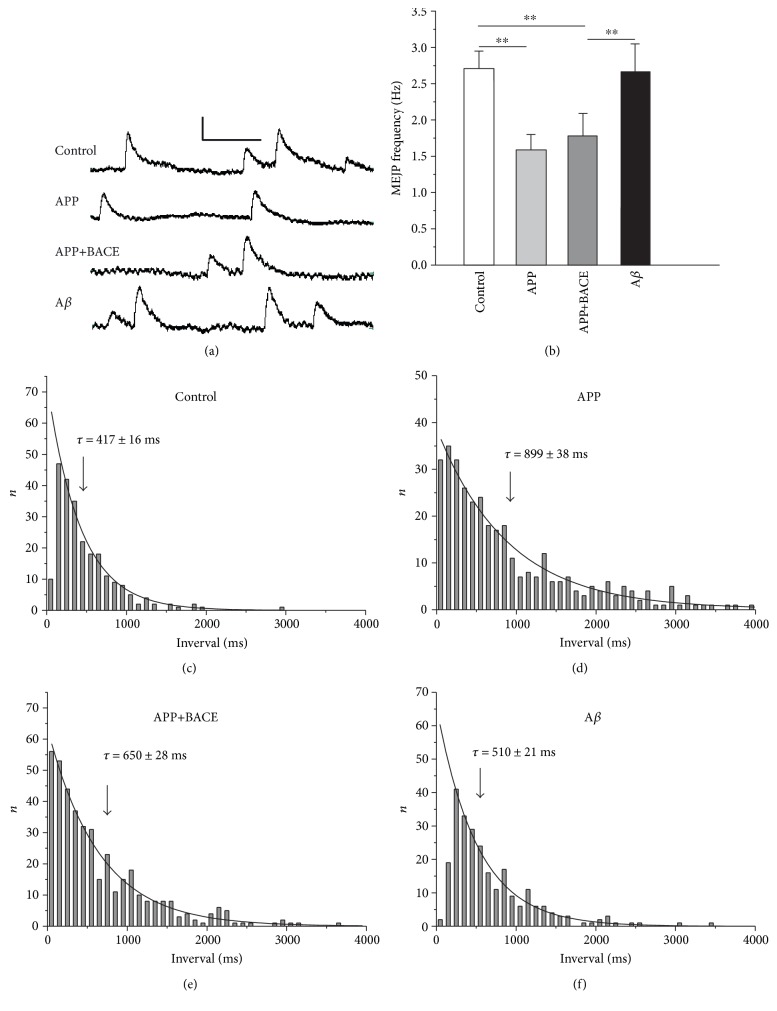
mEJPs recoded at muscle fiber 6 and 7 neuromuscular junctions from transgenic *Drosophila melanogaster* larvae. (a) Sample of original mEJP traces from representative individual experiments. Calibration: vertical, 1 mV; horizontal, 200 ms. (b) Average mEJPs frequency. (c–f) Distribution of inter-event intervals between mEJPs recorded in representative individual experiments. (c) Control, (d) APP, (e) APP+BACE, and (f) А*β* lines. Curve fitting assuming a monoexponential decay confirming the random nature of spontaneous neurotransmitter release in all *Drosophila* lines. *τ*—the time constants, obtained from corresponding monoexponential fitting. Data are presented as mean ± SEM. ^∗∗^*P* < 0.01 compare to corresponding bar.

mEJPs were recorded from muscle fibers 6 and 7 as more convenient for electrophysiology (Figure 5(a)). Previously, it was shown that human *APP* gene expression (in APP and APP+BACE lines) decreases the mEJPs frequency suggesting alterations in synaptic vesicle exocytosis mechanism [27]. To further reveal the background mechanism, in this study, we performed mEJP recordings simultaneously with corresponding confocal imaging and both pre- and postsynaptic mEJPs characteristics were analyzed in detail. Finally, the major novelty of mEJPs recording in this study is the use of A*β* line that provides the possibility to actually separate APP and A*β* effects.

Human *APP* gene expression significantly (*P* < 0.01) decreased mean mEJPs frequency by 41% compared to the control. In the APP+BACE line, mEJPs frequency also was significantly (*P* < 0.01) decreased by 34%. Conversely, in the A*β* line, mEJP frequency was significantly (*P* < 0.01) increased compared to the APP and APP+BACE lines; no difference compared to the control line was observed (Figure 5(b), Table 2).

Spontaneous neurotransmitter release is a random process and as such can be described by a Poisson model. Distribution of mEJPs latencies in all lines were best-fitted with a monoexponential equation predicted by the Poisson model well, thus confirming that under all experimental conditions, the random nature of spontaneous quantal release is not altered. However, as expected from the higher mEJP frequency in the control and A*β* lines, the time constants (*τ*) of distribution were increased in the APP and APP+BACE lines (Figures 5(c), 5(d), 5(e), and 5(f)).

**Figure 6:**
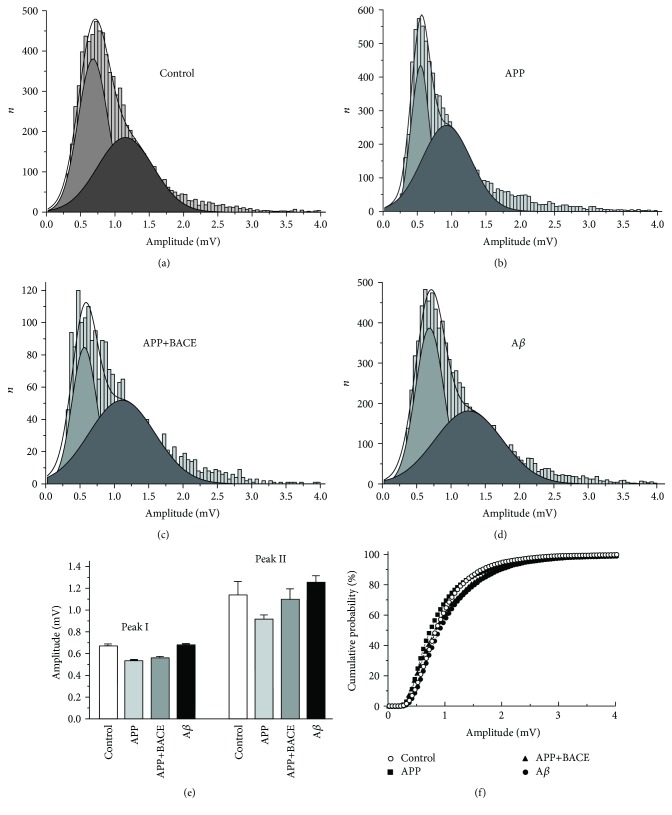
Distribution of mEJPs amplitudes recorded at muscle fiber 6 and 7 neuromuscular junctions from transgenic *Drosophila melanogaster* larvae. (a) Control, (b) APP, (c) APP+BACE, and (d) А*β* lines. Amplitude histograms show similar two-peak nature of mEJPs distributions in all lines (double Gaussian fits are shown). (e) The average amplitude of two peaks from mEJPs amplitude distribution (a–d). (f) Cumulative histogram of the mEJPs amplitude distribution. Total mEJPs number for corresponding distributions (a–d) are included. Data are presented as mean ± SEM.

It was observed that the mean peak amplitude and the rise time and decay time of mEJPs, obtained in the control line, were not significantly changed in APP, APP+BACE, and A*β* lines (Table 2). Notably, the mEJP amplitude distribution in the control line was best fitted by a Gaussian two-peak model with mean peaks at 0.68 mV and 1.15 mV (Figure 6(a)). The ratio of these peaks (1.15/0.68 = 1.69) corresponds well with the observation that quantal size is 53% larger for 1s boutons than for 1b boutons [28]. Accordingly, our data provide the evidence to suggest that “small” and “big” mEJPs in the bimodal distribution were generated from 1b and 1s presynaptic boutons, respectively. Similar bimodal distributions with analogous peaks were obtained in all *Drosophila* lines (Figures 6(a), 6(b), 6(c), and 6(d)) and no pronounced differences from the control line were observed (Figure 6(e)). Cumulative probability curves for amplitude distribution of total mEJPs number also did not differ from the control (Figure 6(f)).

In summary, these results provide evidence that human *APP* overexpression specifically lowers spontaneous neurotransmitter release without essential APP-induced alterations in quantal size and/or postsynaptic events.

## 4. Discussion

AZs represent specialized regions of the presynaptic membrane where quantal neurotransmitter release occurs via synaptic vesicle exocytosis [16, 17]. These highly specific compartments contain synaptic vesicles and serve as a molecular platform for specialized proteins involved in the organization and regulation of neurotransmitter release [18–21]. Accumulated data indicate that APP not only localizes in AZs but also functionally and molecularly interacts with several key proteins of the vesicular exocytosis molecular machinery. Among the APP-associated proteins, a number of components necessary for synaptic vesicle fusion have been identified [22–24]. In particular, altered distribution of a core SNARE complex protein, synaptobrevin, accompanied by disturbances in synaptic vesicle turnover under APP overexpression was observed [12]. It has also been shown that APP altered expression of the calcium sensor synaptotagmin-1 [15]. Moreover, it has been demonstrated that APP localizes to synaptic vesicles in close association with synaptotagmin-1 and might thus play a role in the regulation of synaptic vesicle exocytosis [22].

It has been shown that AZ spacing is regulated by different mechanisms [18, 29]. Our data provide the first evidence that human *APP* gene expression alters AZ distribution, specifically decreasing Brp cluster number per single synaptic bouton including satellite boutons. Notably, *APP* overexpression did not alter total AZ number per NMJ. Consequently, APP-induced decrease of spontaneous neurotransmitter release frequency can be explained by lowered vesicular exocytosis probability rather than by AZ number change.

The role of Brp per se in these phenomena is not clear and remains to be elucidated. Brp is known to be the major structural and functional scaffold protein of the AZ in *Drosophila* [19–21, 25]. This protein is oriented perpendicularly to the AZ membrane and its membrane-proximal N terminus helps to spatial arrangement and clustering of calcium channels. The C-terminus of Brp reaches into the cytoplasm of synaptic boutons to tether and dock synaptic vesicles. In addition, at the *Drosophila melanogaster* NMJ, Brp functionally and molecularly interacts with the calcium sensor synaptotagmin [30]. Taken together, all these features provide evidence to suggest a correlation between Brp spacing and neurotransmitter release probability [19, 31]. Accordingly, at Brp mutant, evoked vesicle release was depressed; however, the mEJPs frequency was not significantly altered [25]. To explain this difference, it should be noted that at *Drosophila*, spontaneous and evoked release are independently regulated at individual AZs [32]. In our experiments, APP-induced Brp loss was accompanied by a significant decrease in spontaneous neurotransmitter release, suggesting the involvement of additional APP-dependent factors. In sum, to gain further insight into the possible interplay between Brp distribution and spontaneous quantal release, special investigations are required.

## 5. Conclusions

Our data demonstrate that human *APP* overexpression enhances synaptogenesis accompanied by altered AZs distribution and lowered spontaneous neurotransmitter release. Presumably, *APP* overexpression specifically disturbs the vesicular exocytosis probability without alteration in the random nature of spontaneous quantal release. These effects are APP specific and do not depend on А*β* production. Taken together, our observation suggests that human *APP* gene expression disturbs both structural and functional organization of AZs resulting in altered spontaneous neurotransmitter release and that these effects are specific for APP.

## Figures and Tables

**Table 1:** Parameters of muscle 4 neuromuscular junctions of transgenic *Drosophila melanogaster* larvae.

Parameters	Control	APP	APP+BACE	A*β*
1b bouton number per NMJ	25 ± 2	49 ± 4^∗∗^	47 ± 3^∗∗^	26 ± 1
Satellite bouton number per NMJ	7.1 ± 0.8	21.6 ± 2.0^∗∗^	12.5 ± 0.7^∗∗^	7.3 ± 0.7
Total bouton number per NMJ	32 ± 2	71 ± 6^∗∗^	60 ± 3^∗∗^	33 ± 2
Brp clusters (active zones) number per 1b bouton	7.2 ± 0.2	4.3 ± 0.1^∗∗^	4.3 ± 0.1^∗∗^	7.5 ± 0.1
Brp clusters (active zones) number per satellite bouton	2.5 ± 0.1	1.7 ± 0.1^∗∗^	1.6 ± 0.1^∗∗^	2.4 ± 0.1
Total Brp clusters (active zones) number per NMJ	174 ± 8	178 ± 12	188 ± 10	194 ± 8
Number of NMJ	36	31	45	59
Number of larvae	9	10	17	26

Data are presented as mean ± SEM. ^∗∗^*P* < 0.01 compared to the control.

**Table 2:** Parameters of mEJPs recoded at muscle 6 and 7 neuromuscular junctions from transgenic *Drosophila melanogaster* larvae.

mEJPs parameter	Control	APP	APP+BACE	A*β*
Peak amplitude (mV)	1.0 ± 0.1	1.0 ± 0.1	1.1 ± 0.1	1.1 ± 0.1
Rise time (ms)	7.5 ± 0.3	9.2 ± 0.4	9.4 ± 0.4	9.2 ± 0.3
Decay time (ms)	29.6 ± 0.8	33.8 ± 0.8	33.4 ± 1.3	34.0 ± 0.9
Frequency (Hz)	2.7 ± 0.2	1.6 ± 0.2^∗∗^	1.8 ± 0.3^∗∗^	2.7 ± 0.4
Number of NMJ	44	30	15	34
Number of larvae	16	12	6	14

Data are presented as mean ± SEM. ^∗∗^*P* < 0.01 compared to the control.
